# Kink-band formation in the directionally-solidified Mg/LPSO two-phase alloys

**DOI:** 10.1080/14686996.2022.2137696

**Published:** 2022-11-03

**Authors:** Toko Tokunaga, Koji Hagihara, Michiaki Yamasaki, Tsuyoshi Mayama, Kazuki Yamamoto, Hiroki Narimoto, Taiki Kida, Yoshihito Kawamura, Takayoshi Nakano

**Affiliations:** a Department of Physical Science and Engineering, Nagoya Institute of Technology, Nagoya, Aichi, Japan; b Division of Materials and Manufacturing Science, Graduate School of Engineering, Suita, Osaka, Japan; c Magnesium Research Center & Department of Materials Science, Kumamoto University, Kumamoto, Japan; d Department of Adaptive Machine Systems, Graduate School of Engineering, Osaka University, Suita, Osaka, Japan

**Keywords:** Mg alloy, strength, LPSO-phase, microstructure, kink band

## Abstract

The variation in the mechanical properties with the volume fraction of the long-period stacking ordered (LPSO) phase in directionally solidified (DS) Mg/LPSO two-phase alloys was examined. Unexpectedly, the yield stress of the DS alloys increases non-monotonically with an increase in the volume fraction of the LPSO phase. The LPSO phase is considered an effective strengthening phase in Mg alloys, when the stress is applied parallel to the growth direction. Nevertheless, the highest strength was obtained in alloys with 61–86 vol.% of the LPSO phase, which was considerably higher than that in the LPSO single-phase alloy. It was clarified that this complicated variation in the yield stress was generated from the change in the formation stress of kink bands, which varied with the thickness of the LPSO-phase grains. Furthermore, the coexistence of Mg in the LPSO phase alloy induced the homogeneous formation of kink bands in the alloys, leading to the enhancement of the ‘kink-band strengthening’. The results demonstrated that microstructural control is significantly important in Mg/LPSO two-phase alloys, in which both phases exhibit strong plastic anisotropy, to realize the maximum mechanical properties.

## GRAPHICAL ABSTRACT

**Figure:**
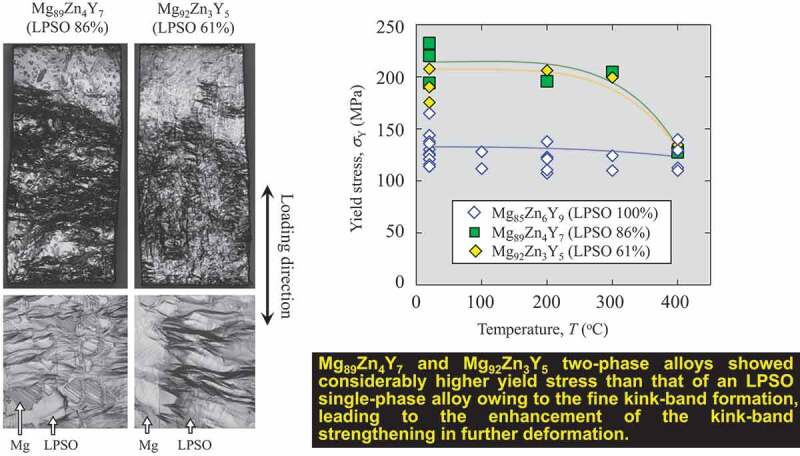

## 1. Introduction

Recently, the importance of developing lightweight high-strength alloys has increased because of increasing awareness of environmental issues and reduction in CO_2_ emissions to prevent global warming. Although Mg alloys are promising candidates owing to their high strength-to-weight ratio, their strength must be improved for use in a wide range of applications [1]. To overcome this disadvantage, the use of the long-period stacking ordered (LPSO) phase has been strongly focused on Mg–Zn–Y ternary alloys [2–21]. It was reported that superior mechanical properties can be achieved in alloys prepared by rapid solidification [2] and thermomechanical processes, such as hot extrusion and hot rolling [3–19]. The strengthening mechanism of these alloys is considered to be strongly related to the strong plastic anisotropy in the LPSO phase [22–24]. The LPSO phase has a closely packed (0001) basal plane with a lengthened stacking sequence along the *c*-axis, typically denoted as 18-fold (18 R) or 14-fold (14 H). In the LPSO phase, Zn/Y atoms are periodically segregated on specific layers [25]. Considering this unique structure, only the basal slip is predominately operative, and the activation of other typical deformation modes observed in Mg, such as the prismatic slip, pyramidal slip, and deformation twin, are strongly hindered, particularly at low temperatures [22–24]. However, a deformation kink band is formed instead of basal slip in relatively wide loading orientations. The formation of kink bands has recently gained attention for use in strengthening the LPSO phase; this is referred to as ‘kink-band strengthening’ [26,27]. Deformation kink bands, in which basal dislocations are aligned nearly along the direction perpendicular to the slip plane, were formed when stress is applied along the direction nearly parallel to the slip plane, resulting in a strain along the *c*-axis. Owing to its geometry, the kink bands strengthen materials since they act as effective obstacles against further motion of the basal dislocations. The variations in mechanical properties of Mg/LPSO two-phase alloy have been reported by some researchers [6,10,28–31], and it has been demonstrated that strength basically increases with the increase in the volume fraction of the LPSO phase. However, the influence of its volume fraction on kink-band strengthening in Mg/LPSO two-phase alloys has not reported so far.

As mentioned, the kink bands form when the stress is applied nearly parallel to the slip plane, where the Schmid factor for the basal slip is negligible. Therefore, to introduce the kink bands in the LPSO phase in order to induce kink-band strengthening, the basal plane in the LPSO phase must be aligned in the loading direction. Thermomechanical processes are known to be effective for controlling the texture. For example, in many of the hexagonal close-packed (hcp) materials, basal planes are aligned along the direction parallel to the applied stress under tensile stress condition. Considering as-cast LPSO single-phase and Mg/LPSO two-phase alloys exhibit poor strengths as low as those of conventional Mg alloys [4,32], it is surmised that thermomechanical processes are essential to activate kink-band strengthening, leading to high-strength LPSO single-phase or Mg/LPSO two-phase alloys. In other words, studies on the strengthening of Mg/LPSO alloys without thermomechanical processes have been less focused.

In the previous study focusing on the LPSO single-phase alloys [24], the directionally solidified (DS) treatment at the slow growth rate of 10 mm/h was found to develop the strong texture in which the (0001) basal plane is aligned parallel to the growth direction without thermomechanical processing. However, there is still no research about the influence of microstructure on the kink-band formation and on the resultant mechanical properties in Mg/LPSO two-phase alloys developed without thermomechanical processes. Therefore, in the present study, to clarify the controlling factors of the mechanical properties of Mg/LPSO two-phase alloys from a basic point of view, the variation in the mechanical properties of Mg/LPSO alloys with the volume fraction of the LPSO phase was examined using DS alloys.

As a result, the experimental results unexpectedly showed that the yield stress of the DS alloys increases non-monotonically with an increase in the volume fraction of the LPSO phase, even though the LPSO phase is considered as an effective strengthening phase in Mg alloys. The origin of this anomalous variation in yield stress is discussed.

## 2. Experimental procedure

Master ingots with five different compositions, Mg_85_Zn_6_Y_9_, Mg_89_Zn_4_Y_7_, Mg_92_Zn_3_Y_5_, Mg_94_Zn_2_Y_4_, and Mg_97_Zn_1_Y_2_ (at%), were prepared by induction melting in a carbon crucible. Directional solidification of the master ingots was conducted using the Bridgman technique in an Ar gas atmosphere at a growth rate of 10 mm/h. The growth rate was determined based on the previous research [24] to achieve the alignment of the basal plane in the LPSO phase grains as described in the previous section.

The microstructures of the alloys were observed by optical microscopy (OM) and scanning electron microscopy (SEM). For OM observations, the surfaces of the specimens were etched with a solution containing 4.2 g of picric acid, 10 ml of distilled water, 70 ml of ethyl alcohol, and 10 ml of acetic acid. The constituent phases in the obtained DS crystals were analyzed by powder X-ray diffraction (XRD) method. The powders for the XRD measurements were prepared by grinding the alloys with an iron file. A magnet was used to remove iron powder that may have contaminated. The texture development in the DS alloys was analyzed by electron backscatter diffraction (EBSD) in the SEM.

To investigate the effect of the volume fraction of the LPSO phase on the mechanical properties, ‘simple compression tests’ were conducted. For the simple compression test, rectangular specimens with dimensions of 2 × 2 × 5 mm^3^ were cut out from the DS crystals by electrodischarge machining. Two loading directions were selected for the simple compression tests; the first and the second loading directions were the parallel direction and the direction inclined at 45° to the growth direction in the DS process, respectively. Hereafter, these directions are denoted the 0°- and 45°-orientations, respectively. Compression tests were performed on an Instron testing machine at a nominal strain rate of 1.67 × 10 ^−4^ s ^−1^ and at room temperature (RT), 200, 300, and 400 °C in vacuum.

**Figure 1.**
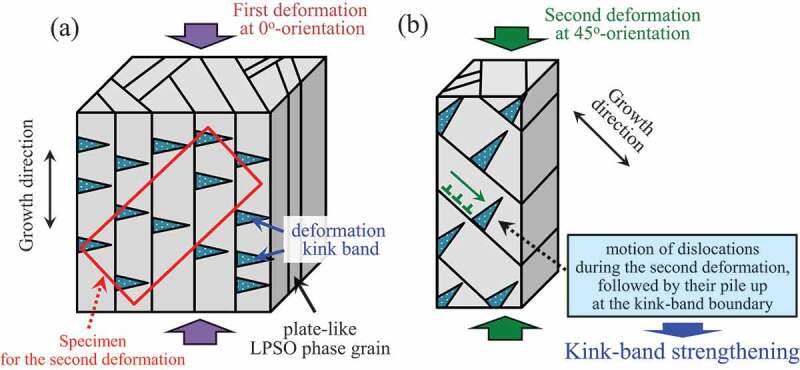
Schematic illustrations showing the double compression test; (a) the first deformation at 0°-orientation, (b) the second deformation at 45°-orientation.

In addition, to elucidate the effect of the kink bands on further deformation, ‘double compression tests’ [27] were conducted as schematically illustrated in Figure 1. First, a rectangular specimen with dimensions of approximately 2 × 5 × 5 mm^3^ was prepared from the DS crystals. The loading direction of the specimen was parallel to the growth direction. The specimen was compressed up to 5% plastic strain at 1.67 × 10^−4^ s^−1^ and at RT, to introduce kink bands; this was called the ‘first deformation’. Thereafter, a specimen with dimensions of approximately 2 × 2 × 5 mm^3^ was cut out from the first deformed specimen such that the compressive loading axis had an angle of 45° to the loading axis of the first deformation. The specimen was again compressed at 1.67 × 10^−4^ s^−1^ and at RT, 200, 300, and 400°C; this was called the ‘second deformation’.

After the simple and the double compression tests, deformation markings on the specimen surfaces were analyzed by OM with Nomarski interference contrast. Furthermore, the variations in crystal orientation due to the deformations were examined by SEM-EBSD.

## 3. Results

### 3.1 Microstructure in the DS alloys

**Figure 2.**
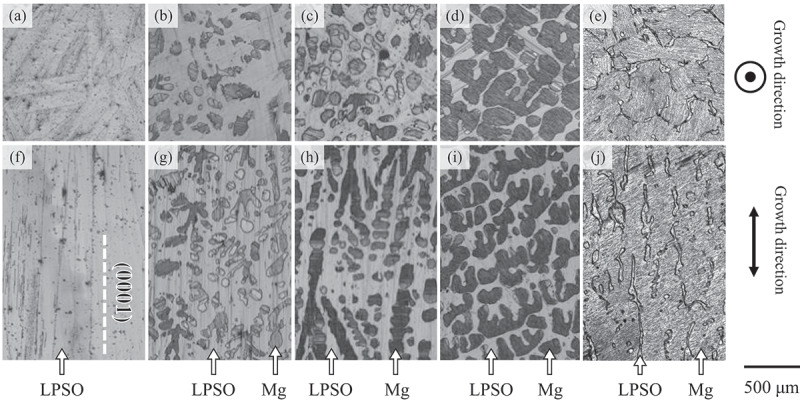
OM images of the microstructures in the as-grown directionally solidified (DS) alloys; (a, f) Mg_85_Zn_6_Y_9_, (b, g) Mg_89_Zn_4_Y_7_, (c, h) Mg_92_Zn_3_Y_5_, (d, i) Mg_94_Zn_2_Y_4_, and (e, j) Mg_97_Zn_1_Y_2_. Figs. (a–e) and (f–j) show the images obtained in the transverse and longitudinal sections, respectively, with respect to the growth direction.

Figure 2 shows the microstructures of the as-grown DS alloys: (a, f) Mg_85_Zn_6_Y_9_, (b, g) Mg_89_Zn_4_Y_7_, (c, h) Mg_92_Zn_3_Y_5_, (d, i) Mg_94_Zn_2_Y_4_, and (e, j) Mg_97_Zn_1_Y_2_. Figures 2(a-e) and 2(f-j) show the microstructures observed in the transverse and longitudinal sections, respectively, with respect to the growth direction. In the alloys with high Mg contents, the hcp-Mg solid solution phase coexisted with the LPSO phase, and the volume fraction of the Mg phase increased as the Mg content increased. The volume fractions of the LPSO phase were measured from the OM images as an area fraction, and estimated to be 100, 86, 61, 39, and 26 vol.% in the Mg_85_Zn_6_Y_9_, Mg_89_Zn_4_Y_7_, Mg_92_Zn_3_Y_5_, Mg_94_Zn_2_Y_4_ and Mg_97_Zn_1_Y_2_ alloys, respectively.

In the Mg_85_Zn_6_Y_9_ LPSO single-phase alloy, the LPSO phase grains exhibited plate-like shapes. In the previous study using the transmission electron microscopy (TEM), the interfaces of the LPSO-phase grains were confirmed to be parallel to the (0001) basal plane [22], and the same feature of the basal plane was confirmed in the Mg/LPSO two-phase alloys by the SEM-EBSD analysis as described later. In the LPSO single-phase alloy, the plate-like LPSO phase grains were well-aligned parallel to the growth direction [Figure 2(b)], though the plate-like grains were randomly aligned on the transverse section with respect to the growth direction [Figure 2(a)]. A similar alignment of the plate-like LPSO-phase grains parallel to the growth direction was observed in the Mg_89_Zn_4_Y_7_ and Mg_92_Zn_3_Y_5_ Mg/LPSO two-phase DS alloys, even in the presence of Mg grains [Figure 2(g, h)]. Considering the above-mentioned characteristics of the microstructure, the average thickness of the plate-like LPSO-phase grains in each alloy could be measured by the OM observation on the transverse section [Figure 2(a-e)]. In the Mg_85_Zn_6_Y_9_ single-phase alloy, the average thickness was evaluated to be approximately 147 μm. As shown in Figure 2, the presence of Mg decreased the thickness of the LPSO-phase grains in the DS alloys. The average thicknesses of the LPSO-phase grains in the Mg_89_Zn_4_Y_7_ and Mg_92_Zn_3_Y_5_ alloys were approximately 64 and 53 μm, respectively. Compared to those alloys, the microstructures were largely different in the Mg_94_Zn_2_Y_4_ and Mg_97_Zn_1_Y_2_ alloys, in which the volume fraction of the Mg phase was higher than that of the LPSO phase. In the alloys, the LPSO-phase grains exhibited network microstructures in the Mg matrix-phase grains.

**Figure 3.**
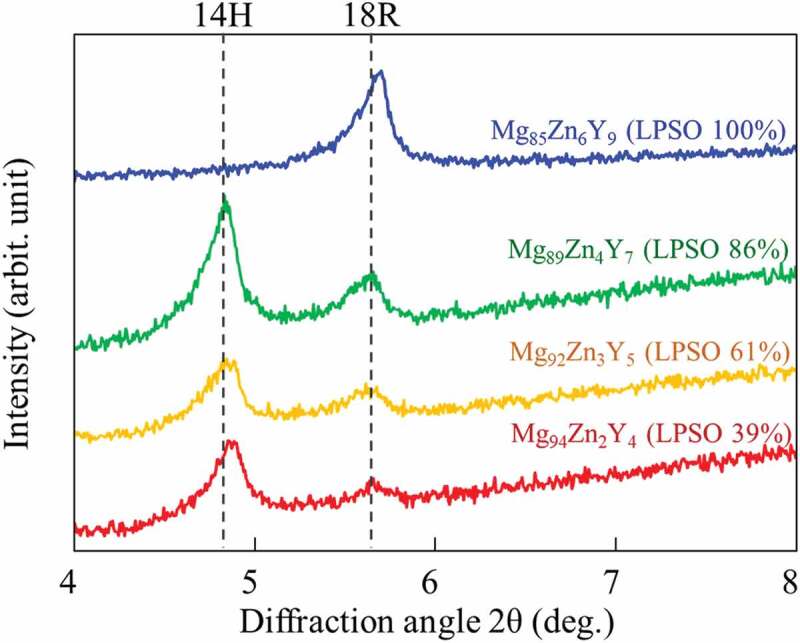
Powder XRD profiles of the Mg_85_Zn_6_Y_9_, Mg_89_Zn_4_Y_7_, Mg_92_Zn_3_Y_5_, and Mg_94_Zn_2_Y_4_ alloys at low diffraction angles below 2θ < 8°.

Figure 3 shows the powder XRD profiles of the alloys measured at low angles. It has been known that the stacking sequence of the (0001) close packed plane in the LPSO phase has some variations, e.g. 18-, 14-, and 10-fold, depending on the alloy composition and heat-treatment condition [22–24]. In the XRD profile taken at high angle [33], since the differences in the positions of diffraction peaks with stacking sequence are small, it is not easy to unambiguously distinguish the diffraction peak position. On the other hand, the difference is clearly distinguished at low angle diffraction [34] because the detected peaks in the low-angle region were derived from (000X) diffraction (X = 6 for 18 R and 7 for 14 H), and the positions only depended on the periodicity of the Zn/Y concentrated layers in the LPSO phase [25]. It is known that the Mg_85_Zn_6_Y_9_ LPSO single-phase alloy comprised only the 18 R-type LPSO phase [24]. Contrarily, in the DS two-phase alloys, the obtained results demonstrated that the crystal structure of the LPSO phases coexisting with Mg was not 18 R, but mainly 14 H. This is in good consistent with the previous reports [23,35]. In Mg/LPSO two-phase alloys, it was reported phase transformation of the LPSO phase occurs from 18 R to 14 H in the annealing at high temperature of 500°C [35], which is owing to the slightly higher Mg content in the LPSO phase (Mg_88_Zn_5_Y_7_) in Mg/LPSO two-phase alloys [22,23] than that in the thermally stable 18 R-LPSO phase (Mg_85_Zn_6_Y_9_) [24]. Because of the slow solidification and cooling process during the DS, the phase transformation is considered to be occurred. It is to note here that in our previous study, no significant difference was observed in the deformation behavior between the 14 H- and 18 R-LPSO phases [22,23]. Thus, it was concluded that the differences in the crystal structure of the alloys in this study did not affect the deformation behavior and were negligible in the following discussion for the deformation.

**Figure 4.**
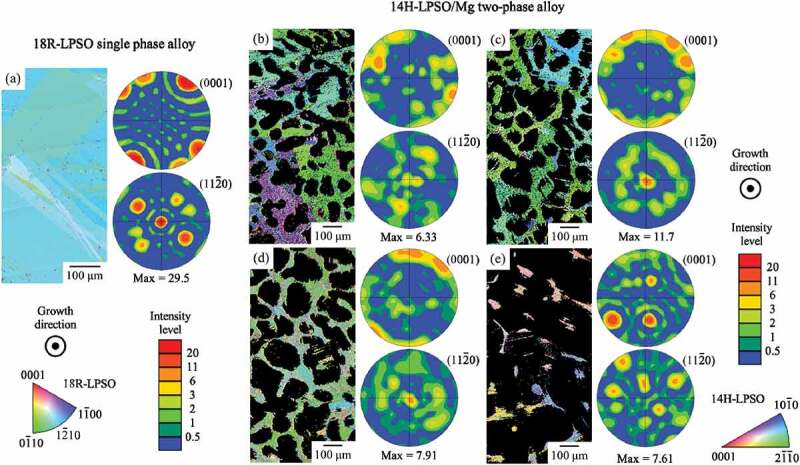
Crystal orientation maps and texture plots of the LPSO phase in the (a) Mg_85_Zn_6_Y_9_, (b) Mg_89_Zn_4_Y_7_, (c) Mg_92_Zn_3_Y_5_, (d) Mg_94_Zn_2_Y_4_, and (e) Mg_97_Zn_1_Y_2_ alloys recorded by SEM-EBSD in the transverse section with respect to the growth direction.

**Figure 5.**
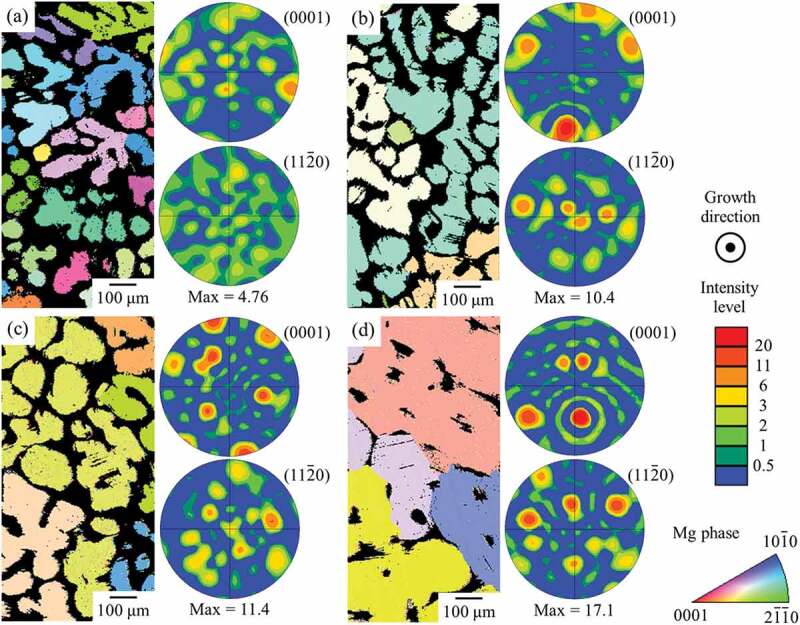
Crystal orientation maps and texture plots of the Mg phase in the (a) Mg_89_Zn_4_Y_7_, (b) Mg_92_Zn_3_Y_5_, (c) Mg_94_Zn_2_Y_4_, and (d) Mg_97_Zn_1_Y_2_ alloys recorded by SEM-EBSD in the transverse section with respect to the growth direction.

Figures 4 and 5 show the crystal orientation maps of the LPSO and Mg phases in the alloys, in the transverse section with respect to the growth direction. In addition, pole figures for (0001) and {11$\overset{ˉ}{2}$0} are also displayed. The crystal orientation maps exhibit the typical part of the field of view. The pole figures were created based on the wider area than the shown orientation maps to obtain statistically correct data. The intensity of the pole figures is indicated as 1 for the completely random case. As shown in Figures 4 and 5, crystallographic texture formation was confirmed in some alloys but its feature was varied with alloy composition. In the Mg_85_Zn_6_Y_9_ LPSO single-phase alloy, [11$\overset{ˉ}{2}$0] was strongly aligned parallel to the growth direction in most of the grains, and [0001] *c*-axis was located perpendicular to the growth direction, as shown in Figure 4(a). This is consistent with the observation results of aligned plate-like shapes of LPSO-phase grain microstructure shown in Figure 2, and also consistent with the previous study, confirming the crystal orientation by TEM analysis [22]. Although, in the two-phase alloys, the preferential alignment of the LPSO-phase grain, in which (0001) is parallel to the growth direction, was not as significant as in the Mg_85_Zn_6_Y_9_ LPSO single-phase alloy, similar crystallographic features were also observed in two-phase alloys. In the Mg_94_Zn_2_Y_4_ alloy, the preferential alignment was weakly remained; however, no preferential alignment was observed in the Mg_97_Zn_1_Y_2_ alloy. The Mg grains exhibited almost random texture in all the alloys in the wide field of view, as shown in Figure 5, although some grains exhibit a similar crystal orientation each other within a narrow region. The specific orientation relationship between the LPSO phase and Mg was not observed in most of the alloys, except for the Mg_97_Zn_1_Y_2_ alloy. In Mg_97_Zn_1_Y_2_ alloy in which the Mg is the predominant constituent phase and the LPSO-phase grains are embedded in the Mg grains, the LPSO-phase grains frequently show the same orientation as the surrounded Mg grains. That is, the LPSO-phase grains in Mg grains frequently showed the crystal orientation relationship of (0001)_Mg_//(0001)_LPSO_ and [11$\overset{ˉ}{2}$0]_Mg_//[11$\overset{ˉ}{2}$0]_LPSO_ as the cast alloys reported in the previous study [21].

### 3.2 Variations in mechanical property and deformation behavior with volume fraction of LPSO phase

**Figure 6.**
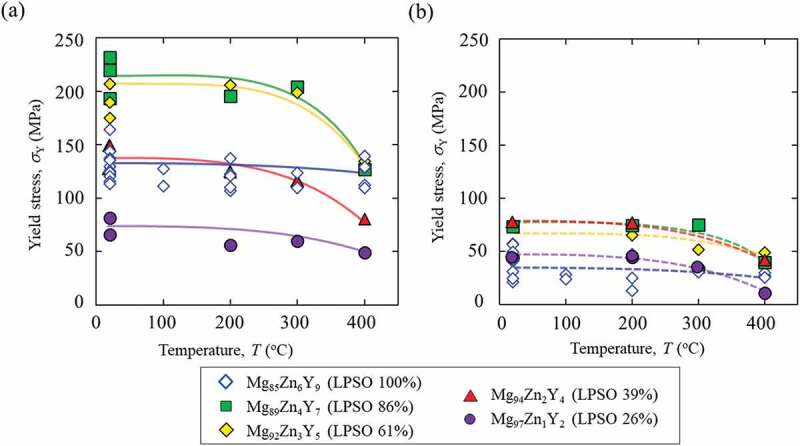
Temperature dependencies of the yield stress of the DS alloys deformed at the (a) 0°- and (b) 45°-orientations.

Figure 6 shows the temperature dependencies of the yield stress (0.2% offset stress) of the DS alloys deformed in the 0°- and 45°-orientations. The deformation behavior of the Mg_85_Zn_6_Y_9_ LPSO single-phase alloy was described in a previous report [24]. The yield stress of the Mg_85_Zn_6_Y_9_ alloy exhibited strong anisotropy. This was attributed to the difference in operative deformation mode. In the 45°-orientation, because the (0001) basal plane in certain LPSO-phase grains was 45°-inclined with respect to the loading axis, the basal slip was predominately operative, resulting in a low yield stress. At the 0°-orientation, a high yield stress of approximately 130 MPa was measured, owing to its negligible Schmid factor for the basal slip, where the (0001) basal plane was parallel to the loading direction. A similar strong anisotropy in the yield stress was observed in other Mg/LPSO two-phase DS alloys. In all the DS alloys, the yield stresses in the 45°-orientation were lower than those in the 0°-orientation, as shown in Figure 6(b).

It must be emphasized here that the yield stress showed complicated variation with the alloy composition. Regarding the yield stress, unexpectedly, the yield stresses of the Mg/LPSO two-phase alloys with compositions of Mg_89_Zn_4_Y_7_ and Mg_92_Zn_3_Y_5_ were considerably higher than those of the Mg_85_Zn_6_Y_9_ LPSO single-phase alloy, even though the LPSO phase is considered as the effective strengthening phase in Mg alloys [3–19], in both of the 0°- and 45°- orientations, and in the wide temperature range between RT and 300 °C.

The variation was especially significant at 0°-orientation. The yield stresses of the Mg/LPSO two-phase alloys with compositions of Mg_89_Zn_4_Y_7_ and Mg_92_Zn_3_Y_5_ were almost 1.7 times higher than those of the Mg_85_Zn_6_Y_9_ LPSO single-phase alloy. The yield stress of the Mg_94_Zn_2_Y_4_ alloy was comparable to that of the Mg_85_Zn_6_Y_9_ LPSO single-phase alloy, and even lower yield stress was only recorded in the Mg_97_Zn_1_Y_2_ alloy. The high yield stresses of the Mg_89_Zn_4_Y_7_ and Mg_92_Zn_3_Y_5_ two-phase alloys were maintained up to 300°C. The yield stresses of the two-phase alloys drastically decreased at 400°C to values comparable with that of the Mg_85_Zn_6_Y_9_ LPSO single-phase alloy.

**Figure 7.**
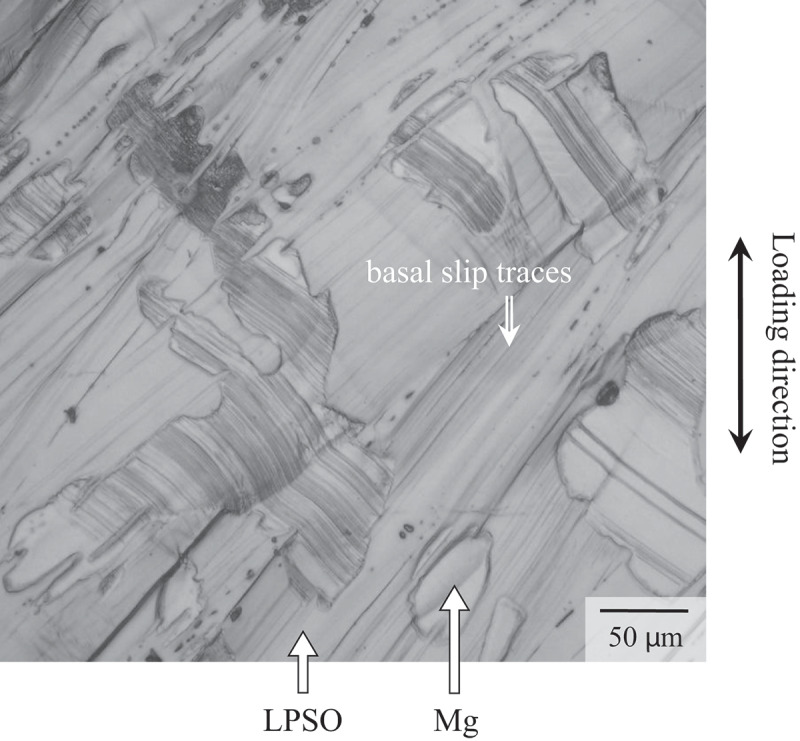
Deformation microstructure of the Mg_92_Zn_3_Y_5_ specimen deformed at the 45°-orientation at RT to 5% plastic strain.

To clarify the origin of strong loading orientation dependence and the anomalous variation in the yield stress with alloy composition, the deformation microstructure was examined. Figure 7 shows the deformation microstructure of the Mg_92_Zn_3_Y_5_ alloy deformed in the 45°-orientation at RT to 5% plastic strain. As shown in Figure 7, significant amounts of slip traces were observed in both the Mg and LPSO-phase grains in the alloys. The deformation microstructure indicated that the basal slips were the controlling mechanism of the deformation in the 45°-orientation, as the same as that observed in the Mg_85_Zn_6_Y_9_ LPSO single-phase alloy [24].

**Figure 8.**
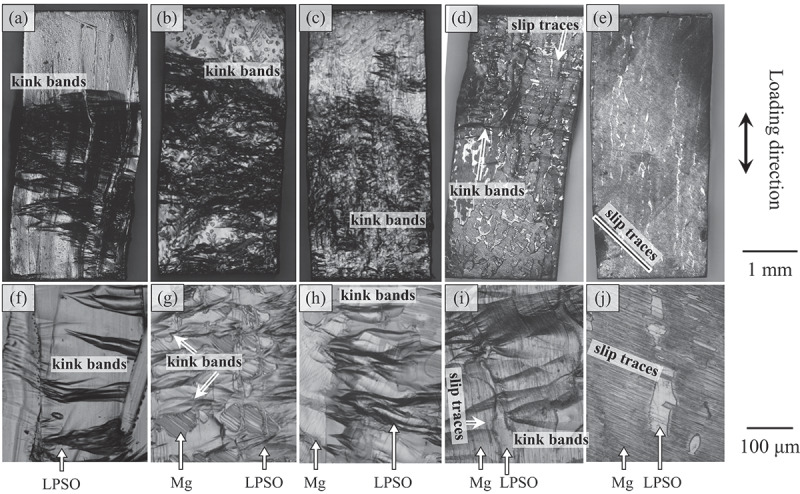
(a–e) Macroscopic views of the specimen deformed at the 0°-orientation at RT to 5% plastic strain and (f–j) high magnification images of the deformation microstructure in (a, b) Mg_85_Zn_6_Y_9_, (c, d) Mg_89_Zn_4_Y_7_, (e, f) Mg_92_Zn_3_Y_5_, (g, h) Mg_94_Zn_2_Y_4_, and (i, j) Mg_97_Zn_1_Y_2_ alloys.

Figures 8(a-e) show the macroscopic view of the specimens deformed in the 0°-orientation at RT in various DS alloys, and Figures 8(f-j) show high magnification images of the deformation microstructure. The deformation microstructure was significantly different from that observed at 45°-orientation. In the Mg_85_Zn_6_Y_9_ [Figures 8(a,f)], Mg_89_Zn_4_Y_7_ [Figures 8(b,g)], and Mg_92_Zn_3_Y_5_ [Figures 8(c,h)] alloys in which the LPSO phase show higher volume fraction, many deformation bands were introduced. Most of the deformation bands were formed nearly perpendicular to the loading axis, i.e. perpendicular to the plate-like shapes of the LPSO-phase grains. In the Mg_94_Zn_2_Y_4_ alloy containing lower volume fraction of LPSO phase than that of Mg phase [Figures 8(d,i)], such deformation bands formation was partly observed in the Mg phase but the amount of them was largely reduced than those in the Mg_85_Zn_6_Y_9_, Mg_89_Zn_4_Y_7_, and Mg_92_Zn_3_Y_5_ alloys. Instead, fine slip traces that correspond to the basal slips were frequently observed. In the Mg_97_Zn_1_Y_2_ alloy [Figures 8(e,j)], the amount of deformation bands was further decreased, and basal slip traces were predominantly observed.

With respect to the morphology, the present deformation bands are generally categorized as twin or kink bands, and they are distinguished by the crystallographic relationship between the bands and matrix. SEM-EBSD analysis was performed to clarify the categories of the deformation bands. It is known that the rotation angle between the inside and outside of the deformation band is fixed in twins, but arbitrary in kink bands. The deformation bands exhibited an arbitrary angle of crystal rotation along an arbitrary crystal rotation axis, which was perpendicular to the (0001) plane. Therefore, the present deformation bands are deformation kink bands as identified in the previous works [36–41]. More detailed analysis method to distinguish kink bands and deformation twins is described in the referred paper [39].

From the observation results of the deformation microstructure, the origin of the significant orientation dependence of the yield stress was found to be derived from the difference in the dominantly operative deformation mode; basal slip at 45°-orientation and formation of kink bands at 0°-orientation, respectively. This variation was induced by the strong texture formation in the LPSO phase, as examined in Figure 4. It was known that the critical resolved shear stress of the basal slip was as low as approximately 10 MPa in the Mg-Y solid solution [42] and also in the LPSO phase [22, 43]. Therefore, the operation of basal slip induces the lower yield stress at 45°-orientation than that at 0°-orientation. In the deformation at 0°-orientation, however, the strong [11$\overset{ˉ}{2}$0] aligned texture hinders the operation of basal slip since its Schmid factor is negligibly small. This induced the much larger yield stress in the 0°-orientation. Therefore, the origin of the orientation dependence of the yield stress observed in Mg/LPSO two-phase DS alloys is basically the same as that clarified in the Mg_85_Zn_6_Y_9_ LPSO single-phase alloy [24].

Regarding another notable feature that the anomalous variation in yield stress with the alloy composition, Mayama et al., very recently reported the similar results in the as-cast alloys, in which basal slip plays a dominant role for deformation owing to their random texture [21]. It was clarified that the Mg/LPSO interfaces act as strong obstacles for slip/twin activity, and induce the anomalous variation in yield stress with the alloy composition in Mg/LPSO two-phase alloys. Since the basal slip is the predominant deformation mode at the 45°-orientation deformation in the present study, similar mechanism must be the origin of the anomalous variation in yield stress in the present two-phase alloys. On the other hand, the anomalous behavior related to the kink-band formation observed in the 0°-orientation has not been studied yet. Therefore, the deformation behavior at 0°-orientation is focused in the following sections.

It is noteworthy that the distributions of the kink bands in the specimen varied significantly depending on the compositions of the alloys, as shown in Figure 8. In the Mg_85_Zn_6_Y_9_ LPSO single-phase alloy, relatively large kink bands were formed locally [Figure 8(a)]. Conversely, small kink bands were homogeneously introduced in the entire specimens of the Mg_89_Zn_4_Y_7_ and Mg_92_Zn_3_Y_5_ Mg/LPSO two-phase alloys [Figures 8(b,c)]. Similar small kink bands were partly observed in the Mg_94_Zn_2_Y_4_ alloy [Figure 8(d)]. However, the amount was much smaller than those in the Mg_89_Zn_4_Y_7_ and Mg_92_Zn_3_Y_5_ alloys. In place of the kink bands, basal slips were observed in certain grains in the Mg_94_Zn_2_Y_4_ alloy. The kink bands were predominately formed in the LPSO phase in the Mg_89_Zn_4_Y_7_ and Mg_92_Zn_3_Y_5_ alloys [Figures 8(g,h)], while they predominately formed in the Mg matrix in the Mg_94_Zn_2_Y_4_ alloy [Figure 8(i)]. It was recently reported that the formation of kink bands in a Mg solid solution is induced by the existence of Zn/Y-segregated stacking fault aggregates, called LPSO nanoplates [20]. In the Mg_97_Zn_1_Y_2_ alloy, few kink bands were observed, and the basal slip in Mg matrix phase dominantly governed the deformation. No significant variation in the deformation microstructure with the test temperature was observed in the range of the present test temperature (RT to 400 °C).

## 4. Discussion

### 4.1 Origin of the anomalous variations in yield stress with volume fraction of LPSO phase

As clarified in Figure 6(a), the yield stress of the Mg/LPSO two-phase DS alloys in the 0°-orientation exhibited a complicated variation with the alloy composition. The yield stresses of the Mg_89_Zn_4_Y_7_ (LPSO: 86 vol.%) and Mg_92_Zn_3_Y_5_ (LPSO: 61 vol.%) alloys were considerably higher than that of the Mg_85_Zn_6_Y_9_ LPSO single-phase alloy, although the LPSO phase is considered as the effective strengthening phase in Mg alloys. The observation of deformation microstructure suggested that kink-band formation must be the controlling mechanism of deformation in the 0°-orientation. Therefore, in this section, the variation in formation behavior of kink bands is focused to elucidate the physical origin of the anomalous variation in yield stress.

Regarding the formation stress of the kink bands, Barsoum *et al* [44–46]. proposed that the remote shear stress, *τ*, which was required to render a kink band nucleus unstable and allow it grow, can be calculated using the following equation, based on a previous study by Frank and Stroh [47]:

(1) $$\tau \;>\sqrt{\frac{4G^{2}b\gamma_{c}}{2\alpha \pi^{2}}\ln\left(\frac{b}{r\gamma_{c}}\right)}$$

where *G* is the shear modulus, *b* is the magnitude of the Burgers vector, *γ*_c_ is the critical kink angle (0.05–5° for most solids), *r* is a value related to the core energy of the dislocation, and 2*α* is the length of a kink band with an elliptical shape. The validity of this equation was quantitatively evaluated by Zhen *et al* [45,46]. with the plastic deformation behaviors of ceramic Ti_3_SiC_2_ and other M_n+1_AX_n_-type phase alloys (MAX-phases). In the LPSO phase, the grains exhibited a unique plate-like shape with an interface parallel to the (0001) plane, corresponding to the slip plane of the basal dislocation. Since kink-band boundaries comprise basal dislocations array, the LPSO-phase grain boundaries can stop the further growth of the kink bands and, the thickness of the grain restricts the length of the kink-band boundary. Thus, the lengths of the kink band, 2*α*, in Equation (1) can be considered to correspond to the thickness of the LPSO-phase grain. Actually, in a previous study using Mg_88_Zn_5_Y_7_ LPSO single-phase DS crystals, it was suggested that the yield stress in the 0°-orientation varied depending on the thickness of the LPSO-phase grain [27]. This demonstrates that the 2*α* corresponds to the thickness of the LPSO phase, and the formation of the kink bands governs the yield of the crystals. To investigate if the present Mg/LPSO two-phase alloys also have the same dependence of the yield stress on the thickness of the LPSO-phase grains and followed the equation, we analyzed the relationship between the yield stress and thickness of the LPSO phase.

**Figure 9.**
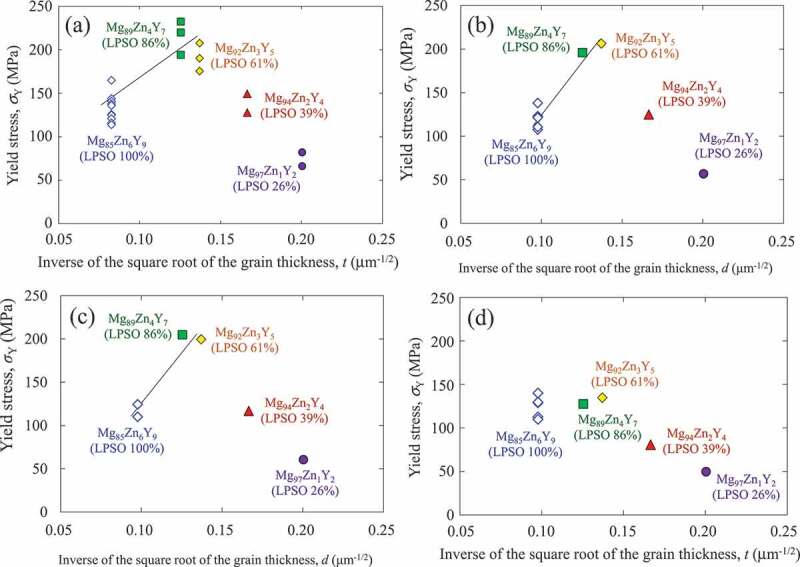
Variations in the yield stress of the DS alloys deformed at the 0°-orientation at test temperatures of (a) RT, (b) 200°C, (c) 300°C, and (d) 400°C, as a function of the inverse of the square root of the average thickness of the LPSO phase grains.

Figure 9(a) shows the variation in the yield stress of the alloys deformed in the 0°-orientation at RT as a function of the inverse of the square root of the average thickness of the LPSO-phase grains. As shown in the results obtained for the Mg_89_Zn_4_Y_7_ and Mg_92_Zn_3_Y_5_ two-phase alloys and the Mg_85_Zn_6_Y_9_ single-phase alloy, the plots were on a straight line, and the yield stress increased with a decrease in the average thickness of the LPSO-phase grains. Thus, it was demonstrated that the relationship between the yield stress and the thickness of the LPSO grain follow Equation (1) . Since the equation represented the relationship between the formation stress and length of kink bands, when the yield stress and the length of the kink bands in the alloy follow the equation, this indicates that the controlling mechanism of the deformation of the alloy is the kink-band formation. This is in good agreement with the observation result of the deformation microstructure [Figure 8]. As shown in Figure 9(b,c), similar relationships were measured in compression tests at 200 and 300°C, but the variation in yield stress with grain thickness of the LPSO phase almost disappeared at 400°C [Figure 9(d)]. This suggests that the controlling mechanism of the yield stress changes from the kink-band formation to something else at 400°C. The contribution of non-basal slips is supposed as a plausible origin of this. However, details have not been elucidated yet, and further studies using TEM have been under consideration.

Contrarily, the Mg_94_Zn_2_Y_4_ and Mg_97_Zn_1_Y_2_ alloys did not follow the equation, as shown in the graph in Figure 9. This implied that the controlling mechanism of deformation was not the kink-band formation ‘in the LPSO phase’ in those alloys. As expected from the deformation microstructure in Figures 8(a-c), the kink-band formation in the LPSO phase was the controlling mechanism of the deformation in the alloys containing over 60% of the LPSO phase. In contrast, other deformation mechanisms, e.g. the basal slip ‘in an Mg solid solution phase’, governed the yield stress of the alloys containing less volume fraction of the LPSO phase than that of the Mg solid solution phase, as observed in the deformation microstructure shown in Figures 8(d,e).

**Figure 10.**
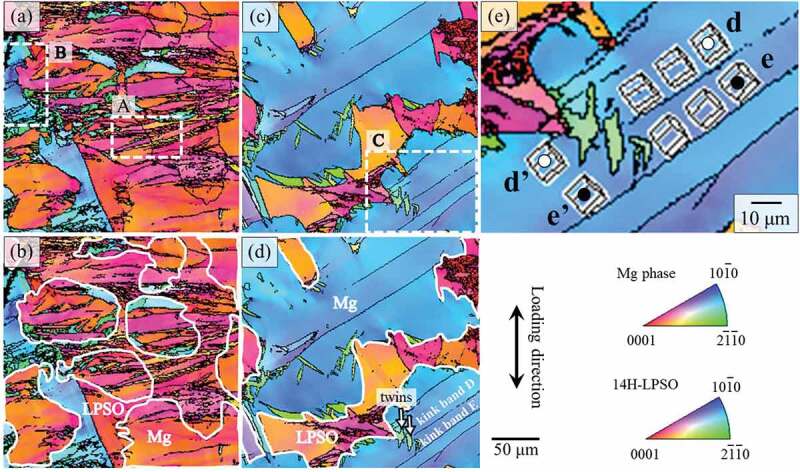
Crystal orientation maps obtained for the specimens deformed at the 0°-orientation at RT to 5% plastic strain. (a, b) Mg_92_Zn_3_Y_5_ and (c–e) Mg_94_Zn_2_Y_4_. (e) enlargement image of the region C in (c).

As demonstrated in Figure 9, the yield stress increased as the thickness of the LPSO phase decreased, accompanied by the increase in the volume fraction of Mg phase up to 40%. To clarify why the increase in the Mg-phase volume fraction did not significantly decrease the yield stress of the Mg/LPSO two-phase alloys, further detailed analysis of the deformation microstructure was conducted by EBSD. Figure 10 shows the crystal orientation maps, focusing on the kink bands. Figures 10(a,b) and (c-e) are obtained from the Mg_92_Zn_3_Y_5_ and Mg_94_Zn_2_Y_4_ alloys, respectively, deformed at RT. In the maps, the boundaries where over 5° of the crystal rotations occurred were indicated by black lines. From observation, and as shown in Figure 8, it was found that the kink bands were predominately formed ‘in the LPSO phase’ in the Mg_92_Zn_3_Y_5_ alloy, while they were predominately formed ‘in the Mg matrix solid solution phase’ in the Mg_94_Zn_2_Y_4_ alloy. In both alloys, the formed kink bands were occasionally propagated beyond the Mg/LPSO interfaces, as indicated in region A in Figure 10(a). However, in other parts, the propagation of the kink bands was stopped at the Mg/LPSO interface, as indicated in region B in Figure 10(a) and region C in Figure 10(c). Figure 10(e) shows an enlarged image of region C. The misorientation angle between the positions d and e, indicated in Figure 10(e), was approximately 10°, while the misorientation angle between d’ and e’ was approximately 3°. This demonstrated that kink band D, indicated in Figure 10(d), was stopped by the protruding LPSO phase, although kink band E was continuously propagated. At the intersection of the kink band and Mg/LPSO interface, {10$\overset{ˉ}{1}$2} deformation twins were frequently observed in the Mg matrix, as indicated by the arrows in Figure 10(d). It was considered that the twins relaxed the stress concentration caused by the kink-band formation and/or hindrance of their propagation at the Mg/LPSO interface.

**Figure 11.**
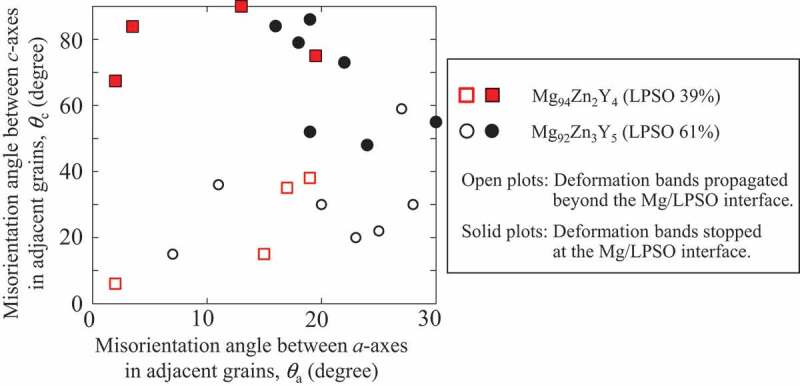
Relationship between the misorientation angle at the Mg/LPSO interface and the propagation behavior of deformation kink bands. The vertical and horizontal axes indicate the misorientation angle of the c- and a-axes in the adjacent Mg/LPSO grains examined by SEM-EBSD.

To clarify whether a kink band penetrated the Mg/LPSO interface or not, the relationship between the misorientation angle at the interface and the penetration frequency of kink bands was examined. As shown in Figure 11, the vertical and horizontal axes indicated the misorientation angles of the *c*- and *a*-axes, respectively, in the adjacent Mg/LPSO grains, as examined by SEM-EBSD. The penetration or nonpenetration of the kink bands occurring at the corresponding two-phase interface is indicated in Figure 11 by open and solid marks, respectively. The results demonstrated that the penetration frequency was strongly affected by the misorientation angle of the *c*-axis between the Mg/LPSO grains. When the misorientation angle of the *c*-axis was higher than 40°, the penetration of kink bands beyond the interface was strongly suppressed, while the misorientation angle of the *a*-axis did not affect the penetration of kink bands. This tendency corresponds with the previous result focused for the influence of LPSO/LPSO grains boundaries measured in the LPSO single-phase alloy [48].

The kink-band boundary is constructed by the alignment of basal dislocations nearly perpendicular to the slip plane. Thus, the misorientation angle of the *c*-axis is the most important factor for the propagation of the kink bands beyond the Mg/LPSO interface. At the Mg/LPSO interface with a large misorientation angle to the *c*-axis, the propagation direction of the kink bands required a large change through the interface, which involved a large stress, leading to the strengthening of the alloy. Thus, to increase the yield strength of the alloy, it is effective to create LPSO/LPSO grain boundaries and/or Mg/LPSO interface with high misorientation angles with respect to the *c*-axes. Here, the introduction of the Mg phase with random texture frequently formed a high misorientation angle at the Mg/LPSO interface with respect to the *c*-axes. Kink bands stopped at such interfaces, which contributed to the high yield stress. This is the reason why the yield stress was almost proportional to the grain thickness of the LPSO phase in Mg_85_Zn_6_Y_9_, Mg_89_Zn_4_Y_7_ and Mg_92_Zn_3_Y_5_ alloys even though the existence of softer Mg solid solution grains.

In the Mg_94_Zn_2_Y_4_ and the Mg_97_Zn_1_Y_2_ alloys that have higher volume fractions of Mg phase than that of the LPSO phase, the formation of the kink band was partly observed in the Mg phase. However, the random texture of Mg phase induced the frequent operation of the basal slip in the Mg matrix phase as shown in Figures 8(d,e), resulting in the large decrease in yield stresses.

### 4.2 Enhancement of the kink-band strengthening in the Mg/LPSO two-phase alloys

In addition to the size, distribution of kink bands varied depending on the alloy composition in the deformed samples. As shown in Figure 8(a), the formation of kink bands was localized in the Mg_85_Zn_6_Y_9_ LPSO single-phase alloy. Conversely, the formation of kink bands was homogeneous in the Mg_89_Zn_4_Y_7_ and Mg_92_Zn_3_Y_5_ alloys, as shown in Figures 8(b,c). This was because the Mg/LPSO interface acted as an effective nucleation site for kink bands via stress concentration. In previous studies, kink-band formation was reported to be induced not only in single-phase alloys, such as the LPSO phase [22–24], MAX phase [37,44–46], and Zn single crystal [36,49], but also in certain two-phase alloys with aligned lamellar microstructures [50–58]. Of those two-phase alloys, homogeneous kink-band formation has been observed in Mg/Mg_17_Al_12_, Mg/Mg_2_Yb, and Al/Al_2_Cu alloys via the introduction of primary grains into the lamellar microstructure [55,56,58]. It was concluded that the primary grains acted as effective nucleation sites for deformation kink-band formation, resulting in their homogeneous distribution in the specimen. The same role was expected for the Mg/LPSO interface in the present alloys, and this was actually achieved. This homogeneous and refined introduction of kink bands is expected to be effective in increasing the strength of the alloys during the additional deformation process, by acting as effective obstacles for the motion of basal dislocations, i.e. ‘kink-band strengthening’ [26,27,59].

**Figure 12.**
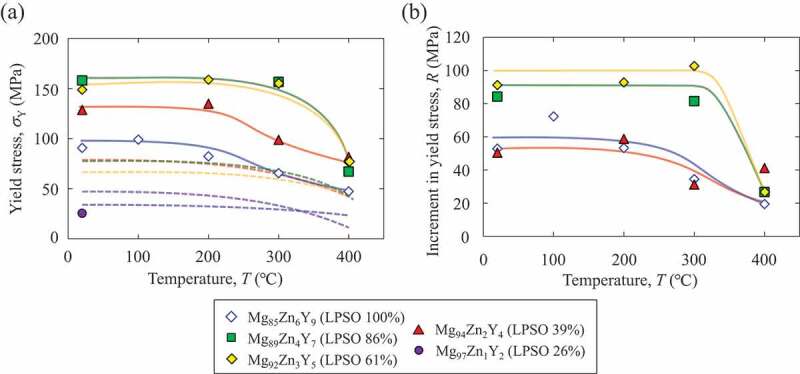
(a) Temperature dependence of the yield stress obtained from the double compression tests. The yield stress in the simple compression test in the 45°-orientation shown in Figure 6(b) is indicated by dotted line, for comparison. (b) Increment in the yield stress by the double compression tests, compared with the virgin DS crystals deformed in the 45°-orientation.

The difference in the degree of kink-band strengthening in each alloy was quantitatively evaluated using double compression tests as explained in Figure 1. The specimen was compressed up to 5% plastic strain at RT to introduce kink bands. Then, a small specimen was cut out from the first deformed specimen such that the second compressive loading axis had an angle of 45° to the loading axis of the first deformation. Figure 12(a) shows the yield stress measured in the double compression tests at various test temperatures. The dotted line in the graph indicates the yield stress measured in the simple compression test shown in Figure 6(b), for comparison. From the results, the increments in the yield stress compared with the virgin DS crystals deformed in the 45°-orientation were evaluated as shown in Figure 12(b). In the Mg_85_Zn_6_Y_9_ LPSO single-phase alloy, the yield stress by the double compression test was significantly higher than that in the simple compression test at the 45°-orientation at all the test temperatures. The result was practically the same as the previous results obtained for the Mg_88_Zn_5_Y_7_ LPSO single-phase alloy [27]. A drastic increase in the yield stress by the double compression tests was observed for the Mg_89_Zn_4_Y_7_ and Mg_92_Zn_3_Y_5_ two-phase alloys. The yield stresses of the double compression tests in these alloys were virtually equal at   150 MPa and were approximately 1.5 times larger than that in the Mg_85_Zn_6_Y_9_ LPSO single-phase alloy at RT. The yield stress in the double compression tests, compared with that in the simple compression tests, also increased in the Mg_94_Zn_2_Y_4_ alloy, in which smaller amounts of kink bands were predominately formed in the Mg phase. Thus, it was demonstrated that kink-band strengthening was effective even in Mg matrix grains. Conversely, yield stress improvement (kink-band strengthening) hardly occurred in the Mg_97_Zn_1_Y_2_ alloy because the kink-band formation did not frequently occur in the first deformation in the 0°-orientation, as shown in Figure 8(e), owing to the less-developed texture both in the LPSO phase and Mg phase.

**Figure 13.**
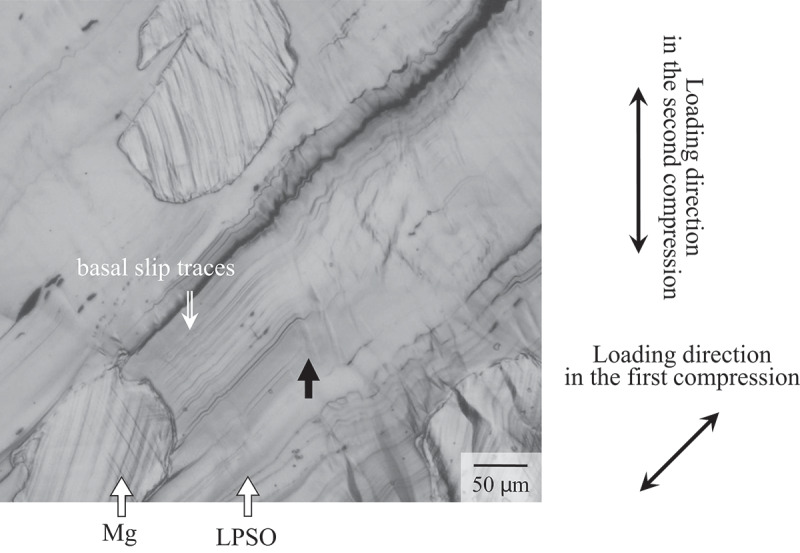
Deformation microstructure of the Mg_92_Zn_3_Y_5_ specimen after double compression test at RT to 2% plastic strain.

In a previous report, we clarified that the yield stress of the Mg_89_Zn_5_Y_7_ LPSO single-phase alloy in the double compression tests can be evaluated using the Hall – Petch-like relationship [27], by regarding the kink-band boundaries as grain boundaries, i.e. as strong obstacles against the motion of basal dislocations. Actually, in the microstructure of the Mg_92_Zn_3_Y_5_ alloy after the double compression test at RT, a typical deformation microstructure after the double compression tests in the Mg/LPSO two-phase alloys, the stoppage of basal slip traces at the kink-band boundaries was observed in the LPSO phase as indicated by the black arrow in Figure 13. The yield stress tended to increase as the distance between the kink-band boundaries decreased. In the Mg_85_Zn_6_Y_9_ single-phase alloy, the distance between the kink bands was roughly estimated to be in the range of 85–500 μm, while it was reduced to 20–70 μm in the Mg_92_Zn_3_Y_5_ Mg/LPSO two-phase alloy. This quantitatively indicates that the two-phase alloy exhibited a more homogeneous distribution of kink bands. Therefore, it was evident that the homogeneous introduction of the kink bands was the origin of the drastic increase in the yield stress in the double compression tests in the Mg/LPSO two-phase alloys. A more quantitative evaluation of the kink-band strengthening to clarify the detailed relation between the kink-band distance and the strength increment, was recently conducted in a separate study on the Mg_99.2_Y_0.6_Zn_0.2_ Mg solid solution single-phase alloy [59].

It should be mentioned that as the origins of the increase in yield stress in the double compression test compared to that in the simple 45° compression test (1) influence of work-hardening by reaction with dislocations introduced during the first deformation, and (2) variation in crystal orientation (Schmid factor) by the kink-band formation, were also supposed in addition to the strengthening by the introduced kink-band boundaries. Regarding (1), in the Mg_97_Zn_1_Y_2_ alloy, in which kink band was not frequently formed, the stress increment is not plotted in Figure 12(b) because the increment was negligible. This indicates that the work hardening induced by the operation of deformation mode other than the kink-band formation is small in the present experimental condition. Regarding (2), if assuming the crystal rotation angle at the kink-band boundary was roughly 15°, the crystal rotation reduces the Schmid factor for basal slip from 0.50 to 0.43 in the ideal condition. This Schmid factor change merely makes the yield stress 1.2 times larger than the ones obtained in the simple compression tests at 45°-orientation. Meanwhile the yield stresses obtained in the present double compression test are more than 2 times. Thus, the kink-band formation as an obstacle against the dislocation motion is considered to the dominant strengthening mechanism in the double compression test, and the strengthening was found to be enhanced in the Mg/LPSO two-phase alloys by the appropriate microstructure control.

In the Mg_85_Zn_6_Y_9_ single-phase alloy, the yield stress in the double compression test maintained a high value of approximately 100 MPa up to 200°C; however, it gradually decreased as the test temperature increased. This was congruent with the results of a previous study on a Mg_88_Zn_5_Y_7_ LPSO single-phase alloy [27]. It was considered that the reduce of yield stress in the double compression test at and above 300°C is related to the activation of a nonbasal slip [22,60]. In contrast, a high yield stress of approximately 150 MPa was maintained up to 300°C in the Mg_89_Zn_4_Y_7_ and Mg_92_Zn_3_Y_5_ two-phase alloys. This implies that the large accommodation of the stress concentration by the nonbasal slip at the kink-band boundaries is more difficult in the Mg/LPSO two-phase alloy, which may be ascribed to the homogenous introduction of many small kink bands. Further studies are required to clarify this hypothesis.

## 5. Conclusion

1. The yield stress of the Mg/LPSO two-phase DS alloys in the 0°-orientation exhibited a complicated variation with the alloy composition. At RT, the yield stresses of Mg_89_Zn_4_Y_7_ (LPSO: 86 vol.%) and Mg_92_Zn_3_Y_5_ (LPSO: 61 vol.%) alloys were considerably higher than that of the Mg_85_Zn_6_Y_9_ LPSO single-phase alloy, although the LPSO phase is considered the effective strengthening phase in Mg alloys. Furthermore, the yield stress of the Mg_94_Zn_2_Y_4_ alloy (LPSO: 39 vol.%) was comparable to that of the Mg_85_Zn_6_Y_9_ alloy. A significantly lower yield stress, compared with that in the Mg_85_Zn_6_Y_9_ single-phase alloy, was only measured in the Mg_97_Zn_1_Y_2_ alloy (LPSO: 26 vol.%).
2. In the Mg/LPSO two-phase alloys, the thickness of the LPSO-phase grain decreased because of the existence of Mg grains. This was the physical origin of the higher yield stress of the Mg/LPSO two-phase alloys compared with that of the LPSO single-phase alloy, in deformation at 0°-orientation where the formation of kink bands carries the strain. The decrease in the thickness of the LPSO phase led to an increase in the formation stress of the kink bands, resulting in an increase in the yield stress of the two-phase alloys.
3. The penetration frequency of the kink band across the Mg/LPSO interface was strongly affected by the misorientation angle of the *c*-axis at the Mg/LPSO boundaries, although the misorientation angle of the *a*-axis was not. The introduction of high misorientation angle boundaries with respect to the *c*-axis was suggested as a strategy to increase the strength of Mg/LPSO two-phase alloys.
4. In the Mg/LPSO two-phase alloys, the kink bands formed more homogeneously during deformation, compared with that in the LPSO single-phase alloy because the Mg grains acted as effective nucleation sites for the kink-band formation. The homogeneous formation of kink bands effectively contributed to the strengthening of the alloys during further deformation, enhancing the “kink-band strengthening” in the Mg/LPSO two-phase alloys.
